# Ovarian cortex freezing as a method of fertility preservation in endometriosis: A case report

**DOI:** 10.1016/j.amsu.2021.103222

**Published:** 2022-01-01

**Authors:** Achmad Kemal Harzif, Gita Pratama, Mila Maidarti, Natasya Prameswari, Amalia Shadrina, Kresna Mutia, Pritta Ameilia Iffanolida, Budi Wiweko

**Affiliations:** a Reproductive Immunoendocrinology Division, Department of Obstetrics and Gynecology, Faculty of Medicine Universitas Indonesia – dr. Cipto Mangunkusumo General Hospital, Jakarta, Indonesia; b Department of Obstetrics and Gynecology, Faculty of Medicine Universitas Indonesia – dr. Cipto Mangunkusumo General Hospital, Jakarta, Indonesia; c Human Reproductive, Infertility, and Family Planning Research Center, Indonesian Medical Education and Research Institute, Faculty of Medicine Universitas Indonesia, Jakarta, Indonesia

**Keywords:** Cryopreservation, Endometriosis, Fertility preservation

## Abstract

**Background:**

Endometriosis affects women in many ways from infertility until reducing ovarian reserve. In women who do not want to immediately conceive, ovarium cortex cryopreservation may be an option for preserving fertility.

**Case presentation:**

Two patients with chief complaints dysmenorrhea and abdominal enlargement, then checked Anti-Mullerian Hormone (AMH) level and Ca-125 level. Patient underwent transrectal ultrasonography, with the result of endometriosis cyst (sized 12 × 9x3 cm and 7 × 10 × 11 cm for first patient, while second patient had 18 × 10 × 14 cm). Then patients underwent cystectomy and ovarian cryopreservation. Histopathology results revealed endometriosis cyst, with different results of follicle density on the healthy cortex. Patient have an AMH level of 1.82 ng/mL before surgery and may decline after surgery. From the AMH normogram, the patient is below the 25th percentile and almost below the 10th percentile, and her biological age is 34. Normal histopathology result of the ovarian cortex suggested that 1.8 to 166 follicles per mm^3^ cortical tissue.

**Discussion:**

We can see from the histopathology examination the density of the follicle was less than normal in this patient. Patients that suffer from endometriosis may have a low ovarian reserve even before surgery. A thorough consultation, followed by ovarian reserve evaluation, disease progression and recurrence of disease are needed to be monitored closely.

**Conclusion:**

From all the methods of fertility preservation, we concluded that this patient is most suitable for ovarian cortex freezing.

## Highlights

• Ovarian cortex is now considered as a source of ovarian tissue preservation.
• Ovarian tissue preservation used to preserve fertility in young women, especially are most at risk of losing ovarian function.
• Patient with minimal to mild disease of endometriomas also reported to have low ovarian reserve.

## 1 Introduction

Endometriosis affects women in many ways as it damages ovarian tissue, leading to infertility and diminished ovarian reserved. Not all women with endometriosis plan to immediately conceive, frequently women came with complaints other than infertility. Diminished ovarian reserve may showed as a result of the damage caused by endometriosis on follicle reservoir, without any surgical interventions [1]. Ovarian endometriosis may appear as endometrioma that can affects fertility by its amounts of free iron, reactive oxygen species, enzymes, and inflammatory molecules leading to diminished ovarian reserve by follicular loss [2,3]. The “Management of Women with Endometriosis” Guideline of The ESHRE on 2013, stated that with endometriomas >3 cm, may be treated for an ovarian cystectomy. Ovarian cystectomy has reported with risk of premature ovarian failure [4]. As we know that ovarian reserve is made of primordial follicles on the outer layer of the ovarian cortex [5]. Mechanisms include excessive removal of healthy ovarian tissues during ovarian cystectomy [3,6,7]. Endometriomas adheres to the surface of the ovary and technical difficulties may ensue in separating the capsule from the healthy ovarian tissue [7]. Patient with low ovarian reserve and suffers from endometriosis may be caused by several factors. For some cases, studies reported that after cystectomy for endometriomas, can cause immediate ovarian failure [1,8]. Some reported that the amount of healthy tissue lost during surgery is larger than the endometriomas itself, also related with electrosurgical and local inflammation caused by the procedure [1,9]. Injury to the blood vessels and stroma resulting reduced in ovarian blood supply post surgery may caused significant decline in ovarian reserve [1]. Diminished anti-Mullerian hormone (AMH), low antral follicle counts, and lower response to ovarian stimulation, may caused by ovarian endometriosis regardless of surgery [1,10]. Age also contributes to oocyte decline, both in number and in quality. Regarding age, it is important to counsel patient about fertility preservation. In preserving fertility, primarily there are three procedures, embryo, oocyte and ovarian tissue preservation. In single women, or women who do not want to immediately conceive, an embryo preservation is not an option. Oocyte preservation is another way of preservation that can be done. Ovarian cortex is now considered as a source of ovarian tissue preservation. Schubert et al. study showed ovarian cortex surrounding ovarian cyst could be considered as a source of ovarian tissue for cryopreservation procedure. The result showed high follicular survival by both histological and viability analysis [11]. Cryopreservation of the ovarian tissue involves a surgical removal of ovarian tissue followed by freezing of the cortical tissue prepared to a thickness of 1 mm. In the beginning, the cortical tissue is processed for freezing that requires cryoprotectants to avoid damage of the cell integrity. The tissue is then stored in a liquid nitrogen, until the tissue is ready to be transplanted. A study of similar survival of follicles were observed in short-term and long-term period [12,13].

Beneath the ovarian surface in the cortex of the ovary, primordial follicles are located. Frozen ovarian tissue can re-establish and ovarian organ function, since it can grow to preovulatory stage. The thickness of the cortex makes it possible for cryoprotectants to penetrate and reduce cell toxic effect. Freezing process needs a programmable freezer that can lower the temperature at a rate that can allow cells to diffuse water, while the organic solvent to enter the cells before vitrification [13,14].

There are two methods for ovarian tissue cryopreservation, slow freezing and vitrification. Vitrification has been successfully applied and have been reported with good result from some studies. Slow freezing is a method of conventional technique, and some reports extensive loss of the follicular pool and excessive damage to stromal cells. Vitrification may be more effective for ovarian preservation, resulting in fewer primordial DNA strand breaks and better stroll cells which may lead to improved tissue function after transplantation. Vitrification cryopreservation is used to avoid formation of ice crystals in ovarian tissue [13,15]. This study has been reported in line with the PROCESS guidelines [16].

## 2 Case presentation

This article showed two women aged 21 years old with chief complaints of dysmenorrhea and abdominal enlargement. Pain was felt before and during the menstrual period, with visual analogue score 8. Both patients were not yet married.

**Fig. 1:**
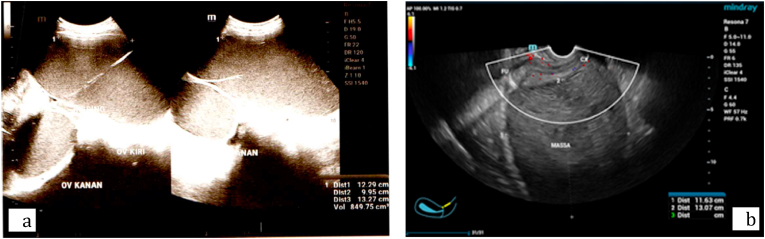
Endometriosis cyst showed on transrectal ultrasound from both patients.

Patient admitted to the gynecology clinic and from physical examination, from rectal examination uterus was not enlarged, cystic mass was felt below the navel and immobile. All patients then were performed transrectal ultrasonography, first patients had ultrasound examination resulted bilateral cystic mass with ground glass appearance sized 12 × 9x3 cm and 7 × 10 × 11 cm, supported by laboratory results with Anti-Mullerian Hormone (AMH) level 1.82 ng/mL and ca-125 level 1188 U/mL. While the second patient had done ultrasound examination resulted right ovarian cystic mass with smooth echointernal sized 18 × 10 × 14 cm, obliterated with douglas pouch, and also bilateral hydronephrosis, the laboratory examination resulted AMH level 2.28 ng/mL and ca-125 level 11244.9 U/mL. The US exam result of both patients were corresponds to endometriosis cyst (Fig. 1).

**Fig. 2:**
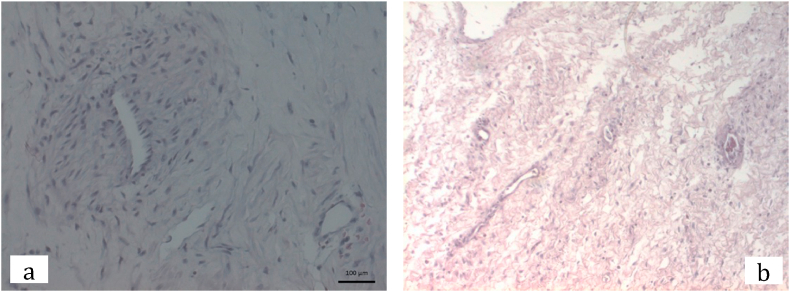
Follicles on the ovarian cortex on histopathology result.

**Fig. 3:**
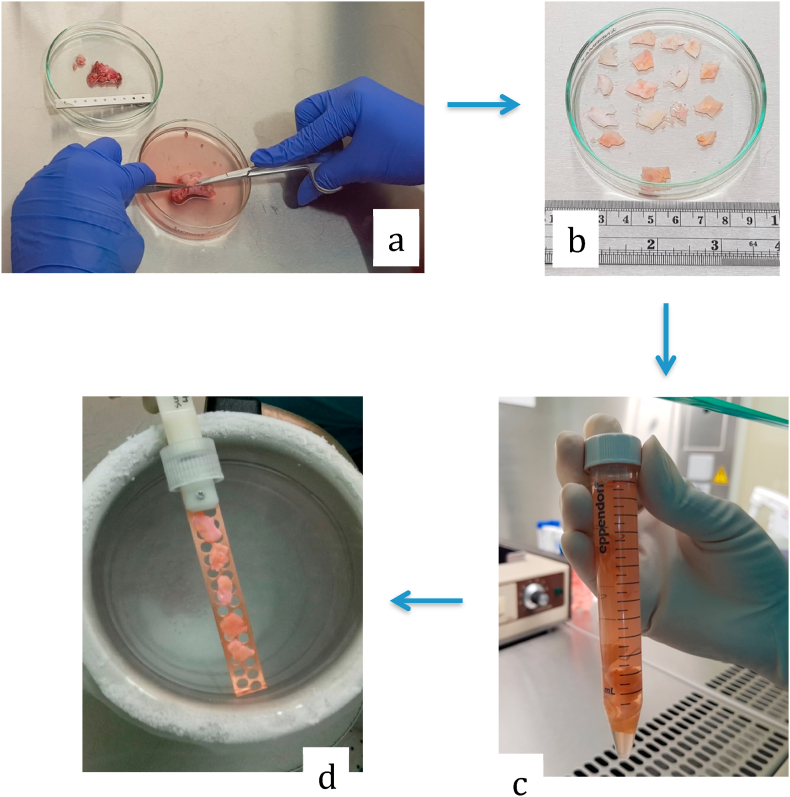
Ovarian cortex preparation by removing the medulla then the cortex was trimmed with thickness of 1–2 mm (a) cut into pieces with size 5 × 5 mm (b) equilibrate in vitrification solution (c) frozen and stored in liquid nitrogen (d).

First patient was then done laparoscopy bilateral cystectomy with ovarian preservation. During the surgery, uterus was within normal limit, found cystic mass with chocolate fluid on both ovaries, sized 12 cm and 15 cm in diameter, then bilateral cystectomy and ovarian preservation were performed (Fig. 3). While second patient then underwent laparotomy cystectomy with ovarian preservation. Intraoperatively seen cystic mass sized 20 cm from left ovary, then procedure continued with cystectomy and ovarian preservation. Histopathology result came out 1 month after both patients did the surgery resulted with correspond to endometriosis cyst. From histopathology we can also see follicles in the ovarian cortex (Fig. 2).

## 3 Discussion

Both patients were diagnosed with a large endometriosis cysts on the ovaries that is necessary for cystectomy procedure. Main concern in these patients was regarding the age, and the fact that the patient was not in immediate concern for having to conceive. Ovarian tissue preservation is currently used to preserve fertility in young women, in some cases who are most at risk of losing ovarian function, such as in these patients, which at risk for losing ovarian function due to endometriosis on the ovaries [1].

The tissue that surrounds endometrioma shows fibrosis formation on the cortical storm and lower follicular density [1,7,17]. This morphological alteration indicates endometriomas are responsible for the low ovarian reserve in patient [[17], [18]]. Endometriosis could also disrupt intrafollicular environment due to reactive oxygen species that can reduce the quality of oocyte [2,[19], [20], [21]]. Oocytes from endometriosis women exhibited cortical granule loss and zona pellucida hardening, that can interfere with fertilization and the ability of embryo to undergo hatching [2].

**Fig. 4:**
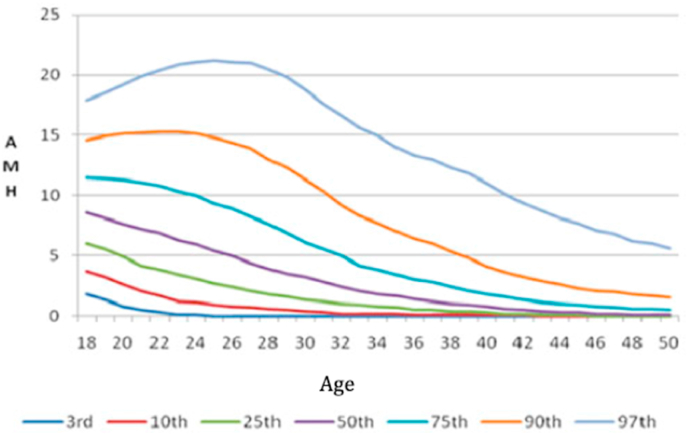
AMH normogram related to age from Wiweko et al. [22].

A meta-analysis by Muzii et al., stated that ovarian reserve evaluated with Anti-Mullerian Hormone (AMH) is reduced on patients with ovarian endometriomas compared to other benign cysts and to patients with healthy ovaries [10]. The AMH level of the first patient was 1.82 ng/mL and 2.28 ng/mL for second patient, which might decline after surgery. However, both patients were a good candidate for fertility preservation. From the AMH nomogram, the patient is below the 25th percentile and almost below the 10th percentile (Fig. 4).

**Fig. 5:**
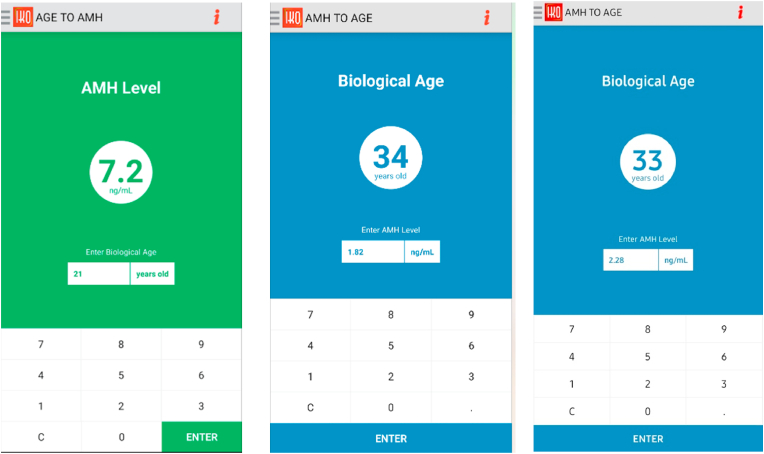
Age to AMH and AMH to age using IKO-calculator.

Using the *Indonesian Kalkulator of Oocytes (IKO)* application, the patients AMH level supposed to be 7.2 ng/mL, and based by her AMH level, the ovarian biological age correspond to 33–34 years old (Fig. 5).

Level of Ca-125 is a peritoneal inflammation marker that is the most commonly described endometriosis biomarker [23]. Ca-125 level in these patients is increased until the highest to 11244.9 U/mL. There are 2 meta-analysis studying about the cut off of Ca-125 for endometriosis, but concluded that Ca-125 is mostly elevated in all stages of endometriosis, but its specificity can be poor because its rise in other gynecological diseases [[23], [24], [25]].

**Fig. 6:**
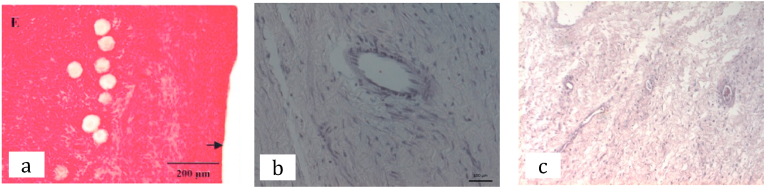
Comparison of the normal ovary showing a cluster of follicles (a) ovarian follicles of first patient (b) ovarian follicles of second patient (c).

Since the patients were not married yet and do not want to immediately conceive embryo or oocyte preservation was not an option. Ovarian tissue preservation was preferred in this patient and was done during the cystectomy procedure. The histopathology result of the ovarian cortex of the normal ovary suggested that 1.8 to 166 follicles per mm3 cortical tissue [25]. In the first patient, we can see from the histopathology examination the density of the follicle was less than normal, which different result was found in the second patient that had more follicles (Fig. 6). Although a small biopsy of ovarian cortex can not always represent the total number of follicles in the ovary, this corresponds to the fact that this patient already has a low ovarian reserve before the surgery [26].

Patient with minimal to mild disease of endometriomas also reported to have low ovarian reserve. In endometriosis there is a focal fibrosis and vascular deficiency in the cortex. Study on primordial follicles in endometriomas are significantly lower together with a concomitant increase in the proportion of non-resting growing follicles [27,28]. This “*Burn-out*” effect on follicle reservoir was shown as major mechanism leading to diminished ovarian reserve, and also a pathway for follicle loss post injury [1,17].

## 4 Conclusion

Patients with endometriosis may have a low ovarian reserve even before surgery, therefore disease progression and recurrence of disease are needed to be monitored closely. From all the methods of fertility preservation, these patients are most suitable for ovarian cortex freezing.

## Ethical approval

This study is exempt from ethnical approval.

## Please state any sources of funding for your research

The authors had no sponsors involved for sources of funding in this article.

## Author contribution statement

Achmad Kemal Harzif: study concept or design.

Gita Pratama: data analysis.

Mila Maidarti: data analysis.

Natasya Prameswari: writing paper.

Amalia Shadrina: writing paper.

Kresna Mutia: data collection.

Pritta Ameilia Iffanolida: data collection.

Budi Wiweko: study concept or design.

## Please state any conflicts of interest

The authors declared have no conflicts of interest to disclose.

## Registration of research studies

Name of the registry: This study was not first in man.

Unique Identifying number or registration ID:

Hyperlink to your specific registration (must be publicly accessible and will be checked):

## Guarantor

The Guarantor was also the corresponding author.

## Consent

Written informed consent was obtained from the patient for publication of this case report and accompanying images. A copy of the written consent is available for review by the Editor-in-Chief of this journal on request.

## Annals of medicine and surgery

The following information is required for submission. Please note that failure to respond to these questions/statements will mean your submission will be returned. If you have nothing to declare in any of these categories then this should be stated.

## Informed consent statement

All the patients provided informed consent to be included in the study.

## Process guidelines

This paper had been in line with PROCESS guidelines [16].

## Provenance and peer review

Not commissioned, externally peer reviewed.

## Declaration of competing interest

None.
